# Screening of Superior Lactic Acid Bacteria from Sourdough and Optimization of Freeze-Dried Bacterial Powder Preparation Technology for Steamed Buns

**DOI:** 10.3390/foods15122130

**Published:** 2026-06-12

**Authors:** Huadi Sun, Lulu Pan, Shunzhi Zhang, Guiting Zhao, Ruixiang Zhao

**Affiliations:** College of Food Engineering, Xinxiang Institute of Engineering, Xinxiang 453700, China; sunhuadi103@126.com (H.S.); october101020@126.com (L.P.); 17837498922@163.com (S.Z.); gtzhao157@163.com (G.Z.)

**Keywords:** mixed fermentation, optimization, lyoprotectant, direct-vat-set starter

## Abstract

To address the industrialization limitations of traditional sourdough and develop high-performance direct-vat-set (DVS) starters for steamed buns, this study screened superior lactic acid bacteria (LAB) and optimized the preparation technology of freeze-dried bacterial powder. The results demonstrated that the compound system of *Lactiplantibacillus plantarum* and *Streptococcus thermophilus* at a 1:1 ratio achieved favorable synergistic fermentation with sourdough, which effectively regulated dough acidification and improved the comprehensive sensory quality of steamed buns. The critical processing parameters, including centrifugation conditions and compound lyoprotectant composition containing trehalose, lactose and skim milk powder, were systematically optimized to significantly enhance the freeze-drying survival ability of compound LAB, and the high fitting degree of the established regression model confirmed the stability and reliability of the optimized formulation. The optimal dosage of freeze-dried DVS powder was verified to rationally modulate dough acidity and comprehensively promote the specific volume, structural characteristics and sensory performance of steamed buns. This systematic study provides robust theoretical and technical support for the industrial application of DVS LAB starters in high-quality steamed bun production, facilitating the modernization of traditional fermented wheat products.

## 1. Introduction

Lactic acid bacteria (LAB) are the most typical and abundant group of microorganisms in traditional sourdough. Accumulating evidence has demonstrated that the synergistic fermentation of sourdough and LAB can improve the texture and flavor of steamed buns, while retarding their staling [1,2]. In recent years, the application of LAB in fermented wheat products has become a research hotspot in the food science field, and existing findings provide a theoretical basis for understanding the application and mechanism of LAB in wheat products. Ma et al. [3] reported that LAB metabolites can effectively improve dough rheological properties, as well as the nutritional components and flavor substances of final products, thereby exerting a significant positive effect on whole-wheat bread quality. Notably, regional differences in the types of starters and dominant LAB strains used in wheat products lead to inconsistencies in research results. Nevertheless, it is well established that complex metabolic pathways among diverse microorganisms in dough produce metabolites such as alcohols, aldehydes, esters, and hydroxyl compounds, which play a crucial role in enhancing the texture and flavor of fermented wheat products. Unlike single sourdough fermentation, multi-strain mixed fermentation facilitates saccharification and esterification, generating a variety of flavor substances such as esters, aldehydes, alcohols, and organic acids that endow steamed buns with unique sensory properties [4]. The effects of different sourdoughs on the rheological properties of steamed bun dough, microstructure of steamed buns, and their steaming quality were researched, and the results showed that sourdough steamed buns had significantly higher quality than conventional ones [5,6]. Collectively, these findings demonstrate the prominent advantages of sourdough-LAB fermentation in improving staple food quality.

However, the theoretical research and industrial application foundation of traditional sourdough remain weak, with its application confined mainly to small-scale workshops and household production, accompanied by several limitations. Firstly, the unavoidable presence of harmful microorganisms poses risks to food safety and hygiene. Secondly, simple production conditions and the lack of independent packaging lead to easy spoilage of steamed buns, making it difficult to maintain product quality and stability during storage. Thirdly, the long fermentation cycle results in low production efficiency, hindering industrialization. Therefore, to promote the sustainable development of traditional sourdough steamed buns, it is essential to retain its merits while addressing the aforementioned drawbacks. Against this backdrop, direct-vat-set (DVS) starters, namely vacuum freeze-dried LAB starters, have been developed. These starters offer advantages such as simplified production processes, improved product quality, and avoidance of poor strain quality caused by insufficient microbial activity. The compound DVS LAB starters were used to ferment coarse grain dough to improve fermentation efficiency and evaluate their effects on dough and steamed bun quality [7]. These reports indicate that DVS LAB starters have great potential to become the future development trend in steamed bun production.

Freeze-drying usually causes severe damage to live cells, resulting in low viable cell counts and rapid viability decline. To mitigate this issue, appropriate lyoprotectants (e.g., sugars, proteins, and amino acids) can reduce physicochemical damage to cells and maintain their activity [5,8]. However, a single lyoprotectant rarely achieves optimal protection effects, and compound formulations of multiple protectants are required to ensure high viable cell counts in freeze-dried bacterial powder. Lee et al. [9] studied the freeze-drying conditions of *L. plantarum* and found that the combination of 10% sorbitol and 10% skim milk powder achieved the highest freeze-drying survival rate (86.37%). These studies confirm the effectiveness of lyoprotectants in protecting cell activity and preventing cell damage, providing a theoretical basis for the development of LAB starters dedicated to steamed buns and facilitating the industrialization of sourdough-LAB mixed fermentation for steamed buns. Despite the well-documented advantages of sourdough-LAB co-fermentation and freeze-dried starter technology, two critical scientific gaps remain. First, most studies focus on single-strain fermentation or empirical sourdough fermentation, while the synergistic mechanisms and optimal strain combinations of multi-LAB compound starters tailored for steamed bun fermentation remain unclear. Second, systematic optimization of compound lyoprotectant formulas and industrial preparation parameters for mixed LAB starters is lacking, and their optimal dosage for steamed bun production is undetermined. Optimized multi-strain LAB compounding can generate synergistic acid-production and flavor-enhancement effects, and rationally designed compound lyoprotectants and processing parameters can sustain high bacterial viability, thereby achieving stable, efficient and high-quality industrial fermentation of sourdough-based steamed buns.

Three representative LAB strains, including *L. plantarum*, *S. thermophilus* and *P. pentosaceus*, were selected for compound fermentation based on their complementary functional characteristics in dough processing. *L. plantarum* possesses strong acid-producing and gluten-modifying capacities; *S. thermophilus* contributes to rapid fermentation and flavor compound synthesis; and *P. pentosaceus* exhibits excellent stress resistance and fermentation stability. The combination of these three strains compensates for the functional deficiencies of single strains, enabling coordinated optimization of dough acidification, structural properties and sensory quality of steamed buns.

This study systematically investigated the effects of different compound LAB starters combined with sourdough on dough fermentation performance and steamed bun quality. Single-factor experiments and response surface methodology were applied to optimize the centrifugation conditions and compound lyoprotectant formula for freeze-dried starter preparation, and the optimal application dosage of the compound DVS starter was finally determined. The novelty of this work lies in the construction of a steamed bun-specific multi-strain compound LAB starter system, the establishment of a targeted industrial freeze-drying preparation process, and the clarification of its optimal application parameters, which effectively addresses the quality instability and low industrial applicability of traditional sourdough fermentation. This work provides a reliable theoretical basis and technical reference for the standardized industrial production of high-quality sourdough-fermented steamed buns and the development of dedicated compound LAB starters for staple food fermentation.

## 2. Materials and Methods

### 2.1. Materials and Reagents

*Lactiplantibacillus plantarum* (GenBank No. MN784485), *Pediococcus pentosaceus* (CGMCC1.7665), and *Streptococcus thermophilus* (CGMCC1.3920) were preserved in the Laboratory of Food Biotechnology, College of Food Science, Henan Institute of Science and Technology. Wheat flour was provided by COFCO Flour Industry Co., Ltd. (Luohe, China). Sourdough powder (Moisture content: 8%, viable bacteria count: >1 × 10^9^, main component: Saccharomyces cerevisiae) was purchased from Angel sourdough Co., Ltd. (Wuhan, China). White granulated sugar was obtained from a local supermarket in Xinxiang City. Sodium chloride and sodium hydroxide were supplied by Tianjin Kemiou Chemical Reagent Co., Ltd. (Tianjin, China). Skim milk powder, trehalose, and lactose were purchased from Henan Huarui Biotechnology Co., Ltd. (Zhengzhou, China). MRS medium was obtained from Beijing Aoboxing Biotechnology Co., Ltd.(Beijing, China). All the aforementioned chemical reagents were of analytical grade.

### 2.2. Instruments and Equipment

DT-501A electronic balance (Changshu Jiaheng Balance Instrument Co., Ltd. Changshu, China); CE6001B small automatic dough mixer (Guangdong Weishida Electrical Appliance Technology Co., Ltd. Foshan, China); PHS-3C pH meter (Shanghai Shengci Instrument Co., Ltd. Shanghai, China); SPX-250 biochemical incubator (Shanghai Yuejin Medical Instrument Factory. Shanghai, China); ZQTY-70N shaking incubator (Shanghai Zhichu Instrument Co., Ltd. Shanghai, China); Xiangyi H2050R desktop high-speed refrigerated centrifuge (Changsha Xiangyi Centrifuge Co., Ltd. Changsha, China); SW-CJ-1D single-person vertical clean bench (Suzhou Zhijing Purification Equipment Co., Ltd. Suzhou, China); Thermo 900 series ultra-low temperature refrigerator (Zhengzhou Jinnouning Instrument Co., Ltd. Zhengzhou, China); and CHRIST ALPHA 1-4 LSC freeze dryer (Christ GmbH, Osterode, Germany).

### 2.3. Activation and Cultivation of Strains

The three LAB strains preserved in a −80 °C refrigerator were taken out for streak culture. A single colony was chosen and inoculated into MRS liquid medium, followed by cultivation in a shaking incubator at 37 °C and 180× *g* for 24 h. The activation was repeated continuously for 2–3 times to obtain activated strains. The activated seed broth was inoculated into MRS liquid medium at an inoculum size of 1% and cultured in a shaking incubator at 37 °C and 180× *g* until the late logarithmic phase for subsequent use.

### 2.4. Preparation of LAB Suspension

Under sterile conditions, 20 mL of LAB broth cultured to the late logarithmic phase was placed into a 50 mL sterile centrifuge tube and centrifuged at 4 °C and 4000× *g* for 15 min. The supernatant was discarded to collect the bacterial pellet, which was washed with sterile normal saline for 2–3 times. An equal volume of sterile water (compared with that before centrifugation) was added and thoroughly mixed to prepare a bacterial suspension for subsequent use (the concentration of the bacterial suspension was adjusted to 4 × 10^9^ CFU/mL); cultivation was terminated when the OD_600_ value of the bacterial suspension reached 0.3–0.5, and the total viable colony count was determined via the plate counting method.

### 2.5. Preparation of Dough and Steamed Buns

#### 2.5.1. Preparation of Dough

A total volume of 20 mL of the bacterial suspension obtained in Section 2.4 was mixed with 80 mL of sterile water, 200× *g* of wheat flour, 2 g of dry sourdough, and 2 g of white granulated sugar in a dough mixer for 10 min until the dough surface became smooth. Dough samples with different starter types were prepared, with the dough without LAB addition serving as the control group. The formulations of different steamed bun dough samples are detailed in Table 1.

#### 2.5.2. Preparation of Steamed Buns

Steamed buns were prepared by a two-step fermentation method with slight modifications [10]. The kneaded dough was first proofed in a constant temperature and humidity environment at 35 °C and 85% relative humidity for 60 min. After primary proofing, the dough was divided into pieces of 80 g each and kneaded vigorously for 5 min to expel internal gas before secondary proofing. The secondary proofing was conducted at 35 °C for 30 min. After proofing, the dough pieces were steamed in boiling water for 20 min. After steaming, they were allowed to stand for 10 min, then taken out, covered with gauze, and cooled to room temperature for subsequent analysis.

### 2.6. Determination of Dough pH and Titratable Acidity (TTA)

During dough fermentation, 10 g of fermented dough was sampled every hour and mixed with 90 mL of deionized water. The mixture was stirred in a magnetic stirrer for 20 min, and the pH value of the dough was then measured. Subsequently, 0.1 mol/L NaOH solution was used for titration until the pH reached 8.5. The volume of NaOH solution consumed was recorded as the TTA of the dough.

### 2.7. Determination of Specific Volume and Height-Diameter Ratio of Steamed Buns

The measurements were performed after the steamed buns were cooled to room temperature as described in Section 2.4, with three replicates for each sample and the average value calculated.

(1) Specific Volume

The cooled steamed bun was weighed to record its mass (m, unit: g). The volume (V, unit: mL) of the steamed bun was determined by the rapeseed displacement method—using a graduated cylinder with a known volume, rapeseeds were filled to a fixed volume, then the steamed bun was placed into the cylinder, and the displaced rapeseed volume was recorded as the steamed bun volume. The specific volume was calculated according to Formula (1):

(1) $$\mathrm{Specific}\;\mathrm{Volume}=\frac{v}{m}$$

(2) Height-diameter Ratio

A vernier caliper was used to determine the maximum height (H, unit: mm) and maximum diameter (D, unit: mm) of each steamed bun. The height-diameter ratio was calculated according to Formula (2):

(2) $$\mathrm{Height}\text{-}\mathrm{diameter}\;\mathrm{Ratio}\;=\;\frac{H}{D}$$

### 2.8. Sensory Evaluation of Steamed Buns

Sensory evaluation was carried out immediately after the steamed buns were cooled to room temperature to avoid sensory deviation caused by moisture loss or temperature change. A professional evaluation panel consisting of 10 trained members (5 males and 5 females, aged 22–45 years) was established, and the evaluation was conducted under natural light with uniform ambient conditions. The sensory panel was trained following the standard GB/T 13868 [11]. Panelists with normal olfactory, gustatory, visual (including color vision), and tactile perception were preliminarily screened. A three-point test was further applied to exclude panelists with low sensory sensitivity and inconsistent judgment. Repeated blind sample evaluation and in-group discussion calibration were performed to eliminate individual descriptive deviations. Monthly repeatability tests were implemented, and variance analysis was used to screen and remove abnormal evaluation data. Targeted training focused on the unique sensory characteristics of steamed buns, including tactile properties (texture resilience and adhesion) and hot-aroma attributes (yeast flavor and sour odor).

The sensory evaluation indicators included Epidermal color (10 points), Epidermal structure (15 points), Internal structure (15 points), Elasticity (15 points), Taste (30 points), and Flavor (15 points) with a total score of 100 points. The specific evaluation criteria are detailed in Table 2. Each panel member evaluated the samples blindly (coded with random numbers) to eliminate subjective bias, and each sample was evaluated in triplicate. The final sensory score of each sample was the average value of all evaluation results, and the data were statistically analyzed.

### 2.9. Selection of Centrifugation Conditions for LAB

The LAB culture was mixed thoroughly when it reached the end of the logarithmic growth phase. An amount of 25 mL of the culture was placed in a 50 mL centrifuge tube and centrifuged at 4 °C. The centrifugation was conducted at 4000, 6000, and 8000× *g* for 5, 10, and 15 min, respectively. The supernatant was discarded and the bacterial sediment was collected. An equal volume of physiological saline was added and mixed thoroughly. The total number of viable bacteria was calculated by the plate count method, and the centrifugal recovery rate was computed according to Formula (3):

(3) $$\mathrm{Centrifugation}\;\mathrm{yield}(\%)=\frac{\mathrm{The}\;\mathrm{viable}\;\mathrm{bacteria}\;\mathrm{count}\;\mathrm{of}\;\mathrm{the}\;\mathrm{bacterial}\;\mathrm{sludge}\;\mathrm{mixed}\;\mathrm{with}\;\mathrm{physiological}\;\mathrm{saline}\;}{\mathrm{The}\;\mathrm{viable}\;\mathrm{bacteria}\;\mathrm{count}\;\mathrm{of}\;\mathrm{the}\;\mathrm{bacterial}\;\mathrm{liquid}\;\mathrm{before}\;\mathrm{centrifugation}}\times 100\%$$

The optimal centrifugation conditions for LAB were determined based on the viable count and recovery rate.

### 2.10. Preparation of Freeze-Dried Bacterial Powder

The freeze-dried bacterial powder was prepared following the process of strain activation → expanded cultivation → centrifugation for bacterial pellet collection → addition of lyoprotectant → subpackaging and pre-freezing → vacuum freeze-drying.

The activated LAB strains were subjected to expanded cultivation in MRS liquid medium under the conditions of 37 °C and 180× *g* until the late logarithmic phase. The bacterial broth was then centrifuged under the optimal conditions determined in Section 2.9 to collect the bacterial pellet, which was washed with sterile normal saline for 2–3 times to remove medium residues. Subsequently, a compound lyoprotectant was added to the bacterial pellet at a volume of 1/10 of the original bacterial broth, and the mixture was thoroughly stirred to form a uniform bacterial suspension.

The prepared bacterial suspension was subpackaged into sterile freeze-drying vials with equal volume per vial and placed in an ultra-low temperature refrigerator at −80 °C for pre-freezing for 4 h until completely solidified. Then, the pre-frozen samples were transferred to a CHRIST ALPHA 1-4 LSC freeze dryer for vacuum freeze-drying, with the cold trap temperature set at −70 °C and vacuum degree maintained at 1 Pa, and the drying process lasted for 24 h.

After freeze-drying, sterile normal saline with the same volume as the original bacterial broth was added to the vials for rehydration, and the mixture was gently shaken and rehydrated for 30 min. The total number of viable bacteria per unit volume before and after freeze-drying was determined by the plate counting method, and the freeze-drying survival rate was calculated according to Formula (4):

(4) $$\mathrm{Freeze}\text{-}\mathrm{drying}\;\mathrm{survival}\;\mathrm{rate}(\%)=\frac{\mathrm{The}\;\mathrm{number}\;\mathrm{of}\;\mathrm{viable}\;\mathrm{bacteria}\;\mathrm{in}\;\mathrm{the}\;\mathrm{bacterial}\;\mathrm{liquid}\;\mathrm{after}\;\mathrm{freeze}\text{-}\mathrm{drying}}{\mathrm{The}\;\mathrm{number}\;\mathrm{of}\;\mathrm{viable}\;\mathrm{bacteria}\;\mathrm{in}\;\mathrm{the}\;\mathrm{bacterial}\;\mathrm{liquid}\;\mathrm{before}\;\mathrm{freeze}\text{-}\mathrm{drying}}\times 100\%$$

### 2.11. Single-Factor Experiments

Four common lyoprotectants (trehalose, lactose, skim milk powder, and sorbitol) were selected to investigate their protective effects on LAB during freeze-drying, with the freeze-drying survival rate as the evaluation index.

(1) Effect of trehalose concentration: Bacterial pellets were collected and mixed with 0.5 mL of trehalose solutions at mass fractions of 4%, 8%, 12%, 16%, and 20%, respectively. The additions of lactose, skim milk powder, and sorbitol were 0, and the group with normal saline added was used as the control. Vacuum freeze-drying was performed, and the freeze-drying survival rate was calculated according to Formula (4).
(2) Effect of lactose concentration: Bacterial pellets were collected and mixed with 0.5 mL of lactose solutions at mass fractions of 9%, 12%, 15%, 18%, and 21%, respectively. The additions of trehalose, skim milk powder, and sorbitol were 0, and the group with normal saline added was used as the control. Vacuum freeze-drying was performed, and the freeze-drying survival rate was calculated according to Formula (4).
(3) Effect of skim milk powder concentration: Bacterial pellets were collected and mixed with 0.5 mL of skim milk powder solutions at mass fractions of 4%, 8%, 12%, 16%, and 20%, respectively. The additions of trehalose, lactose, and sorbitol were 0, and the group with normal saline added was used as the control. Vacuum freeze-drying was performed, and the freeze-drying survival rate was calculated according to Formula (4).
(4) Effect of sorbitol concentration: Bacterial pellets were collected and mixed with 0.5 mL of sorbitol solutions at mass fractions of 3%, 5%, 7%, 9%, and 11%, respectively. The additions of trehalose, lactose, and skim milk powder were 0, and the group with normal saline added was used as the control. Vacuum freeze-drying was performed, and the freeze-drying survival rate was calculated according to Formula (4).

### 2.12. Response Surface Design

Based on the single-factor experimental results, trehalose (A), lactose (B), and skim milk powder (C) were selected as independent variables, and the freeze-drying survival rate (Y) was used as the response value. A three-factor three-level Box-Behnken design was adopted to optimize the lyoprotectant formula. The levels of the factors are shown in Table 3.

### 2.13. Selection of Addition Level of LAB Powder

With the dry sourdough addition fixed at 1%, the effects of different LAB powder addition levels (0.5%, 1%, 1.5%, 2%, 2.5%) on dough pH and TTA during fermentation, as well as the specific volume, height-diameter ratio, and sensory score of steamed buns, were determined. The determination methods of dough pH and TTA, specific volume and height-diameter ratio of steamed buns, and sensory evaluation were the same as those described in Section 2.6, Section 2.7 and Section 2.8, respectively. The optimal addition level of LAB powder was comprehensively evaluated based on these indicators.

### 2.14. Evaluation Storage Stability of Freeze-Dried Bacterial Powder

The lyophilized bacterial powder was transferred into sterilized brown glass bottles and stored at 25 °C for 90 days, followed by the determination of viable cell count, moisture content, and cell survival rate.

### 2.15. Data Analysis

All statistical analyses were performed using Design-Expert and SPSS 22.0 software. Prior to analysis, the normality of data distribution and homogeneity of variance were verified to satisfy the prerequisites of one-way analysis of variance (ANOVA). All experiments were conducted in three independent biological replicates, with each replicate containing two technical measurements. After significant differences were detected by ANOVA (*p* < 0.05), Tukey’s multiple range test was applied for pairwise post hoc comparisons to identify differences among treatments. All significant difference letters presented in figures and tables were generated based on Tukey’s test. Data were expressed as mean ± standard deviation. Statistical significance was defined at *p* < 0.05 for all analyses.

## 3. Results

### 3.1. Effects of Different LAB Starters on Dough pH and TTA

**Figure 1:**
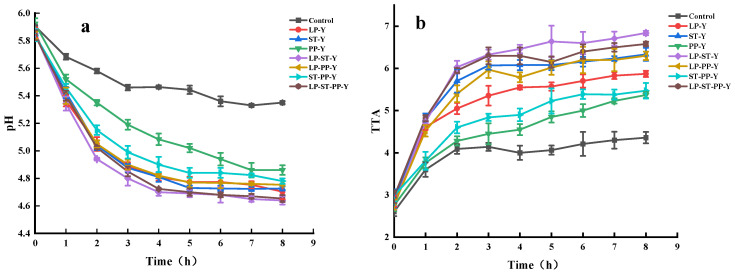
Changes in pH (**a**) and TTA (**b**) of dough during fermentation with different lactic acid bacteria starter cultures. Note: Control is sourdough addition 1%.

Figure 1 shows how co-fermentation of sourdough with different LAB starters affects dough pH and TTA. The incorporation of LAB lowers dough initial pH. For the control sample, dough pH declines gradually from 5.91 to 5.35 throughout fermentation. By contrast, dough fermented with sourdough-LAB blends undergoes a sharp pH drop over the initial 0–4 h before plateauing from 4 to 8 h. Dough TTA rises rapidly and then levels off, mirroring the changing pattern of pH. This phenomenon arises because LAB catabolize dough carbohydrates in early fermentation to synthesize organic acids; accumulating acids reduce ambient pH and progressively elevate TTA.

Acid production varies markedly among tested LAB strains. *L. plantarum* and *S. thermophilus* generate significantly more organic acids than *P. pentosaceus* (*p* < 0.05). In comparison with single-strain fermentation, mixed LAB cultures exhibit superior acid-forming ability. Among all treatments, the LP-ST-Y group achieves the most prominent shifts in pH and TTA, reflecting accelerated dough acidification via multi-strain co-fermentation.

*P. pentosaceus* features slow proliferation and a prolonged growth cycle, restricting organic acid buildup over identical fermentation periods and leading to weaker acid production relative to *L. plantarum* and *S. thermophilus*.

### 3.2. Effects of Different LAB Starters on Specific Volume and Height-Diameter Ratio of Steamed Buns

**Figure 2:**
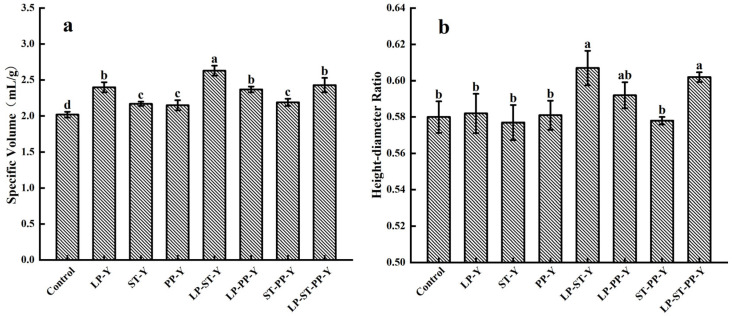
Effects of different lactic acid bacteria starters on the specific volume (**a**) and height-diameter ratio (**b**) of steamed buns. Note: Control is sourdough addition 1%; different letters in the figure indicate significant differences between values (*p* < 0.05).

Figure 2 presents the influences of various starter formulations on steamed bun specific volume and height-to-diameter ratio. Relative to the control, co-fermentation with sourdough and LAB markedly improves specific volume, with the LP-ST-Y group reaching the maximum value at a 26.13% elevation versus control. The LP-ST-Y, LP-PP-Y and LP-ST-PP-Y treatments yield significantly higher height-to-diameter ratios (*p* < 0.05), whereas remaining groups show no statistical difference (*p* > 0.05). Such improvements stem from sourdough and LAB metabolites: microbial carbohydrate catabolism generates CO_2_ to expand dough volume, while by-products including glycerol and succinate strengthen dough gas-holding capacity.

### 3.3. Effects of Different LAB Starters on Sensory Score of Steamed Buns

**Figure 3:**
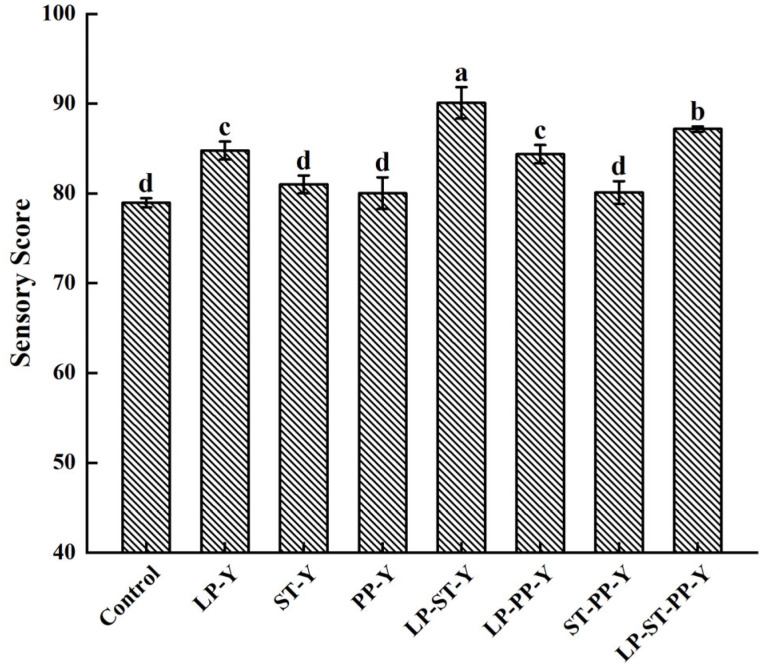
Effects of different starters on sensory score of steamed buns. Note: Control is sourdough addition 1%; different letters in the figure indicate significant differences between values (*p* < 0.05).

The sensory scores directly reflect consumer acceptability of steamed buns, as summarized in Figure 3. Treatments follow the descending sensory ranking: LP-ST-Y > LP-ST-PP-Y > LP-Y, LP-PP-Y > ST-Y, PP-Y, ST-PP-Y, control. The LP-ST-Y group achieved the highest score of 90.09, representing a 14.10% improvement over the control (78.96). These results confirm that co-fermentation of *L. plantarum*, *S. thermophilus* and sourdough effectively upgrades steamed bun sensory quality.

By evaluating dough pH and TTA during fermentation, together with the specific volume, height-to-diameter ratio and sensory performance of finished steamed buns, the LP-ST-Y group exhibited the optimal overall fermentation performance. These findings confirmed that the combined fermentation of *L. plantarum* and *S. thermophilus* with sourdough is the most suitable starter system for the subsequent preparation and investigation of freeze-dried LAB powder.

### 3.4. Determination of Optimal Centrifugation Conditions

Table 4 summarizes LAB recovery under varied centrifugation regimes. Recovery reaches the minimum (58.09%) at 4000× *g* for 5 min, owing to insufficient centrifugal force that fails to fully sediment bacterial cells. Conversely, overhigh centrifugal force triggers severe mechanical injury and cell mortality. Balancing cell sedimentation and post-centrifugation viability is therefore essential. The highest recovery (97.17%) is obtained at 6000× *g* for 15 min, differing significantly from other treatments (*p* < 0.05). Accordingly, 6000× *g* and 15 min are selected as the optimal centrifugation parameters.

### 3.5. Single-Factor Experiments Results

**Figure 4:**
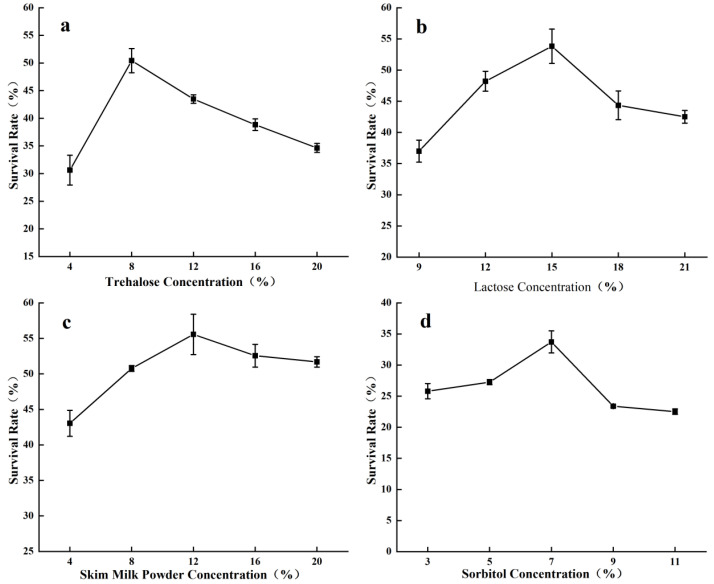
Effect of trehalose (**a**), lactose (**b**), skim milk powder (**c**), and sorbitol (**d**) concentration on freeze-drying survival rate of LAB.

Figure 4a illustrates the influence of trehalose concentration on LAB freeze-drying survival. Survival increased first and then declined with increasing trehalose dosage. The lowest survival rate (30.63%) occurred at 4% trehalose, while the maximum value (50.43%) was achieved at 8%. Appropriate trehalose concentrations fill protein dehydration gaps and form a stable glassy protective layer, effectively preserving cell activity. In contrast, excessive trehalose produces an overly rigid glassy matrix, distorts cellular structures, and reduces viability. Therefore, 8% was determined as the optimal trehalose concentration.

Figure 4b presents the protective effect of lactose. Similarly, lactose improved cell survival in a concentration-dependent manner before inducing a decline. A significant increase in survival was observed at lactose concentrations of 9–15%, with the highest survival rate (53.82%) obtained at 15%, confirming the effective lyoprotective performance of lactose. Higher lactose concentrations elevated solution osmotic pressure, causing cellular dehydration and cell death, thereby decreasing survival.

Figure 4c shows the effect of skim milk powder. Increasing skim milk powder concentration significantly promoted cell survival, peaking at 12% with a viability of 55.56%. Further concentration increases generated excessive osmotic stress, leading to cell dehydration and a gradual reduction in freeze-drying tolerance.

Figure 4d displays the protective performance of sorbitol. Cell survival increased to a maximum of 33.72% at 7% sorbitol and decreased thereafter. High sorbitol concentrations created an extreme osmotic environment incompatible with cell metabolism, resulting in membrane dehydration and structural damage. Although moderate sorbitol provided certain protection, its overall efficacy was limited.

Single-factor results demonstrated that trehalose, lactose, and skim milk powder exhibited markedly superior lyoprotective effects compared with sorbitol, which can be attributed to their distinct molecular protective mechanisms. Accordingly, these three agents were selected for subsequent response surface optimization.

### 3.6. Optimization of Lyoprotectant Formula by Response Surface Methodology

Based on single-factor findings, response surface methodology (RSM) was adopted to optimize the composite lyoprotectant formulation for mixed LAB. Experimental data are listed in Table 5. A three-factor, three-level central composite design was implemented using trehalose (A), lactose (B) and skim milk powder (C) as independent variables, with freeze-drying viability (Y) of mixed LAB as the response value. Regression fitting was conducted via Design-Expert 8.0 software, yielding the following quadratic polynomial regression model:

Y = 84.93 + 10.73A − 3.22B + 4.44C − 4.49AB + 8.20AC + 7.09BC − 15.45A^2^ − 9.94B^2^ − 12.16C^2^

Wherein, Y is the freeze-drying survival rate of LAB, and A, B, C represent trehalose, lactose, and skim milk powder, respectively.

**Table 5:** Results of response surface methodology experiments.

Experiment No.	A: Trehalose/%	B: Lactose/%	C: Skim Milk Powder/%	Y: Survival Rate/%
1	1	1	0	62.56
2	0	0	0	83.86
3	1	−1	0	77.48
4	−1	0	−1	48.19
5	0	0	0	85.99
6	0	1	1	68.98
7	−1	1	0	50.59
8	−1	0	1	44.50
9	0	0	0	87.72
10	0	0	0	81.60
11	1	0	1	82.86
12	0	1	−1	49.74
13	0	−1	1	61.74
14	−1	−1	0	47.55
15	0	0	0	85.47
16	1	0	−1	53.74
17	0	−1	−1	70.88

Model reliability was evaluated via analysis of variance (ANOVA) and F-test, with results summarized in Table 6. The model determination coefficient R^2^ = 0.9872 and adjusted R^2^_adj_ = 0.9707 demonstrate satisfactory fitness and reliable predictive capacity for mixed LAB freeze-drying viability. The high model F-value of 59.80 (*p* < 0.0001) confirms extreme statistical significance, whereas the non-significant lack-of-fit F = 1.89 (*p* = 0.2723) verifies adequate model fit. Significance analysis of regression coefficients reveals the linear term A (trehalose) is highly significant (*p* < 0.0001), B (lactose) is significant (*p* < 0.05), and C (skim milk powder) is highly significant (*p* < 0.01). All pairwise interactions of AB, AC and BC reach statistical significance (*p* < 0.05). According to corresponding *F*-values, the influencing sequence of the three variables on freeze-drying viability ranks as follows: A (trehalose) > C (skim milk powder) > B (lactose).

### 3.7. Analysis of Interaction Effects of Response Surface Factors

**Figure 5:**
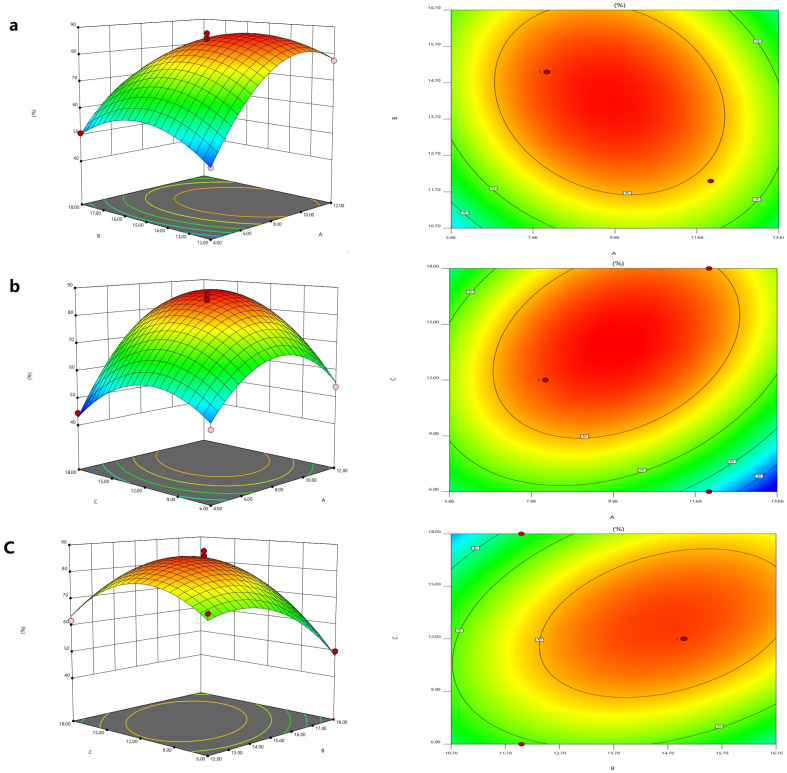
Effects of trehalose (**a**), lactose (**b**), skim milk powder (**c**) concentrations on the freeze-drying survival rate of lactic acid bacteria. Note: (**a**) Interaction between trehalose and lactose; (**b**) interaction between trehalose and skim milk powder; (**c**) interaction between lactose and skim milk powder.

The curvature of response surfaces and contour spacing intuitively reflect the sensitivity of LAB freeze-drying viability to each variable. A steep surface denotes pronounced viability variation against dosage changes, whereas a gentle slope implies low factor sensitivity. Figure 5a–c presents three-dimensional response surfaces and corresponding contour plots for pairwise factor interactions, plotted with the third variable fixed at its central coded level.

In Figure 5a, viability rises then declines with increasing trehalose and lactose dosages. The elliptical contour shape confirms a significant AB interaction (*p* < 0.05). Denser contours along the trehalose axis reveal that trehalose exerts a stronger influence than lactose. For Figure 5b, co-varying trehalose and skim milk powder produce an initial rise followed by a fall in viability; the saddle-shaped 3D surface and elliptical contours verify a significant AC interaction (*p* < 0.05). In Figure 5c, the more elongated elliptical contours for lactose and skim milk powder indicate a prominent BC interaction (*p* < 0.05). Denser contours and steeper surface gradients along the skim milk powder direction demonstrate its greater impact relative to lactose. Collectively, contour distribution and surface steepness rank factor show importance as trehalose > skim milk powder > lactose, in good agreement with ANOVA outcomes.

### 3.8. Prediction and Verification of Optimal Experimental Conditions

The regression model predicts an optimal composite lyoprotectant formulation consisting of 9.19% trehalose, 14.61% lactose and 13.16% skim milk powder, corresponding to a theoretical freeze-drying viability of 87.88%. For practical manufacturing convenience, the formulation was rounded to 9.20% trehalose, 14.60% lactose and 13.20% skim milk powder for experimental validation. Triplicate verification yields an actual viability of (88.67 ± 0.92)%, only 0.90% deviated from the predicted value. This close consistency confirms the reliability and practicability of the RSM-optimized lyoprotectant recipe.

### 3.9. Determination of Optimal Addition Level of LAB Powder

#### 3.9.1. Effects of Different LAB Powder Addition Levels on Dough pH and TTA

**Figure 6:**
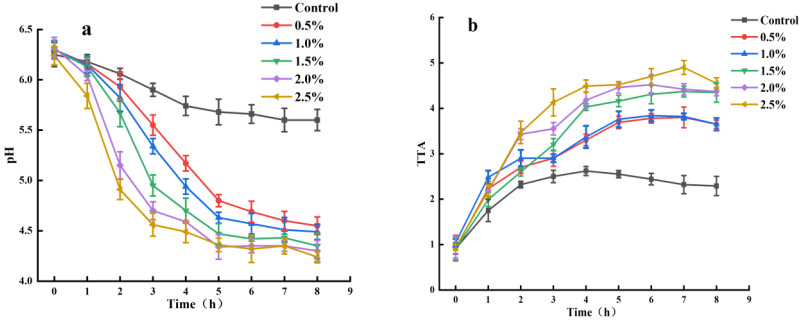
Effects of different LAB powder addition levels on pH (**a**) and TTA (**b**) variations of fermented dough. Note: Control is sourdough addition 1%.

Figure 6 depicts dough pH and TTA dynamics at varied LAB powder supplementation levels. The control group displays gradual pH reduction and slow TTA elevation throughout fermentation. In contrast, dough incorporated with LAB powder undergoes markedly accelerated pH decline and TTA accumulation. At 0.5% and 1% LAB addition, dough pH falls steadily across the whole fermentation period; for 1.5–2.5% supplementation, pH drops sharply in early fermentation and plateaus after 5 h, synchronizing with corresponding TTA variations.

Added LAB accelerates dough acidification and organic acid accumulation via microbial metabolism. Nevertheless, acidification must be moderately regulated for steamed bun manufacturing. Excessively rapid acidification triggers excessive gluten hydrolysis, impairing gas retention, restricting loaf expansion and inducing dull, yellowish crust. The optimal final dough pH ranges from 4.5 to 5.0, enabling balanced co-fermentation between sourdough and supplemented LAB. Accordingly, 0.5% and 1% are determined as the optimal LAB powder dosages.

#### 3.9.2. Effects of Different LAB Powder Addition Levels on Specific Volume and Height-Diameter Ratio of Steamed Buns

**Figure 7:**
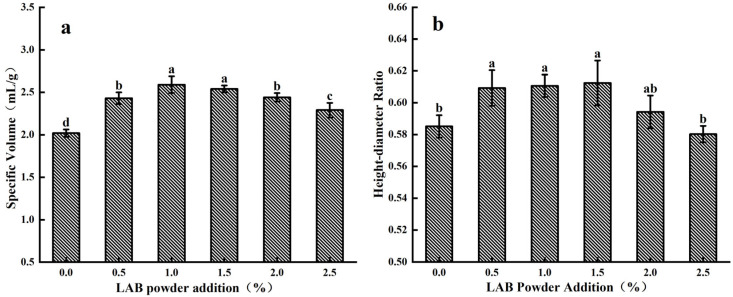
Effects of different LAB powder addition levels on specific volume (**a**) and height-diameter ratio (**b**) of steamed buns. Note: Different letters in the figure indicate significant differences between values (*p* < 0.05).

Figure 7 illustrates how varying LAB powder dosages affect steamed bun specific volume and height-to-diameter ratio. Samples co-fermented with sourdough and LAB present significantly superior indicators versus the control (*p* < 0.05), peaking at 1% and 1.5% supplementation. Sourdough metabolism generates CO_2_ to expand dough volume; meanwhile, LAB-derived mild acidic surroundings facilitate sourdough proliferation, and metabolites including glycerol and succinate optimize dough rheology and gas retention. By contrast, raising LAB dosage to 2.5% reduces both parameters drastically. Excessive organic acid secretion triggers over-acidification, over-relaxing gluten networks, deteriorating gas-holding ability and ultimately causing dough shrinkage and collapse. In conclusion, 1–1.5% LAB powder is optimal for maximizing steamed bun specific volume and height-to-diameter ratio.

#### 3.9.3. Effects of Different LAB Powder Addition Levels on Sensory Score of Steamed Buns

**Figure 8:**
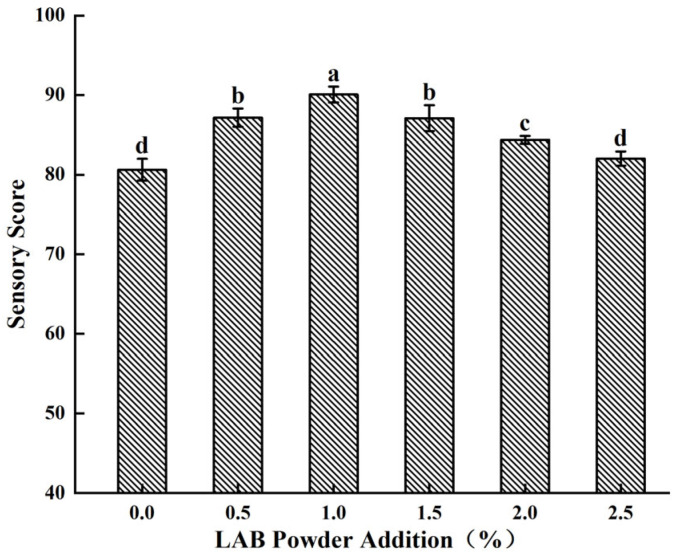
Effects of different lactic acid bacteria powder addition levels on the sensory score of steamed buns. Note: Different letters in the figure indicate significant differences between values (*p* < 0.05).

Figure 8 presents the sensory scores of steamed buns supplemented with different concentrations of LAB powder. The sensory quality of steamed buns follows a clear descending order based on LAB addition levels: 1.0% (90.09 ± 1.00) > 0.5% (87.17 ± 1.15) > 1.5% (87.11 ± 1.63) > 2.0% (84.38 ± 0.48) > 2.5% (82.03 ± 0.90) > 0.0% (80.62 ± 1.37). Steamed buns with 1.0% LAB powder addition exhibited a 11.75% higher sensory score than the control group, demonstrating that the synergistic fermentation of 1% LAB powder and sourdough achieves the best sensory improvement effect.

Combining the comprehensive evaluation of dough pH, TTA, specific volume, height-to-diameter ratio, and sensory performance, the optimal LAB powder dosage was determined. With a fixed 1% dry sourdough addition, supplementation of 1% LAB powder endowed dough with moderate acidity. This treatment significantly improved the specific volume, height-to-diameter ratio, and overall sensory quality of steamed buns compared with the control. Consequently, 1% was identified as the optimal addition level of the prepared compound LAB powder for steamed bun fermentation.

### 3.10. The Influence of Storage Conditions on Freeze-Dried Bacterial Powder

The physicochemical and microbiological stability of optimized freeze-dried LAB powder was evaluated after 90 days of storage at 25 °C by measuring viable cell count, moisture content, and cell survival rate. Resuluts were showed in Table 7. Freshly prepared powder showed superior initial quality, with a viable cell count of 4.0 × 10^9^ CFU/g, moisture content of 1.8%, and a cell survival rate of 95%. After ambient storage, the viable cell count slightly decreased to 3.3 × 10^9^ CFU/g, while moisture content marginally increased to 2.1%, corresponding to a reduced cell survival rate of 78%.

## 4. Discussion

During dough fermentation, pH reflects hydrogen ion concentration and TTA represents dough sourness; both are key indicators of fermentation performance, directly affecting dough structure and final product quality [12]. In the early fermentation stage, microorganisms rapidly consume nutrients, gradually reducing the acid-producing capacity of lactic acid bacteria (LAB) in the later stage, which stabilizes pH and TTA values. This trend is consistent with the findings of Cardinali et al. [13], confirming that mixed fermentation of sourdough and LAB facilitates the production and accumulation of organic acids. Among the tested strains, *L. plantarum* exhibits the fastest growth rate and strongest acid-producing ability in sourdough, while *L*. *casei*, *L*. *paracasei*, and *P*. *pentosaceus* show slower proliferation and weaker acid-producing capacity [14]. The outstanding performance of the mixed starter can be attributed to the positive synergism between *L. plantarum* and *S. thermophilus*. *L. plantarum* exhibits strong adaptability in dough matrices and a high acid-producing capacity, which rapidly regulates dough acidity and optimizes the gluten network structure. Meanwhile, *S. thermophilus* is capable of producing abundant flavor-related metabolites and cooperating well with *L. plantarum* during fermentation. When co-cultured, the two strains complement each other in growth and metabolic patterns: their combined action further promotes the accumulation of organic acids, enhances gas retention capacity of dough, and endows steamed buns with preferable texture and richer aroma. Notably, the specific volume of steamed buns increased by 26.13% compared with the control group, which was closely associated with the dough acidification rate driven by the co-culture of *L. plantarum* and *S. thermophilus*. The two strains exhibited a moderate and continuous acidification rate during fermentation. The gradual decline in dough pH activated endogenous proteases in a mild manner, moderately degraded gluten and optimized the gluten network structure. A well-organized gluten network effectively trapped carbon dioxide produced by microbial metabolism, thereby significantly enhancing dough expansion capacity and ultimately leading to the remarkable rise in specific volume. Excessively fast acidification would cause over-degradation of gluten and impair gas retention, while insufficient acid production could not effectively improve dough rheological properties. Therefore, the appropriate acidification rate achieved by the selected LAB combination was the key factor responsible for the prominent improvement in specific volume. Compared with single-strain fermentation, this co-culture system presents more stable fermentation performance and better comprehensive effects on improving the overall quality of final products, which explains why this combination was selected as the optimal starter in the present study. Previous studies on dough co-fermented with single or multiple LAB strains and sourdough reported that LAB supplementation promotes organic acid accumulation, with multi-strain fermentation yielding the highest lactic and acetic acid contents [15]. These findings align with our results, demonstrating that multi-strain mixed fermentation exerts superior acid-producing effects. LAB metabolites can improve the gluten network by promoting glutenin cross-linking, forming a dense and stable structure that enhances gluten extensibility and CO_2_ retention, thereby increasing dough specific volume and height-diameter ratio [16]. Xu et al. [17] and Wang et al. [18] reported that mixed fermentation of LAB and sourdough significantly enhances Saccharomyces cerevisiae-mediated CO_2_ production, improving bread volume and texture. Similarly, our results showed that adding *L. plantarum*-fermented sourdough improves steamed bun sensory scores and specific volume, confirming that sourdough-LAB mixed fermentation positively impacts steamed bun volume development.

Consistent with Sha et al. [5], LAB supplementation further optimizes gluten network structure and enhances steamed bun texture and sensory attributes. The acidic environment induced by LAB fermentation activates proteases, degrades glutenin, improves dough rheological properties, and imparts desirable color, texture, elasticity, and taste to steamed buns. Additionally, lactic acid generated during mixed fermentation reacts with alcohols, ketones, and other compounds to produce characteristic flavor compounds, significantly enhancing steamed bun aroma and taste [19]. Dough components including starch, protein, lipids, cellulose, and minerals, supplemented with appropriate sucrose, provide sufficient carbon, nitrogen, and mineral sources for LAB growth and metabolism. Application of LAB starters in dough fermentation yields abundant organic acids, conferring a unique flavor profile that cannot be achieved by single sourdough fermentation alone [20,21]. For vacuum freeze-drying, lactose can adhere to the cell membrane surface to form a protective layer, reducing intracellular ice crystal formation and preserving cell integrity [22,23]. In this study, lactose supplementation enhanced LAB freeze tolerance and significantly improved freeze-drying survival rate, leading to the optimal lactose concentration of 15% [24]. Gul et al. [25] optimized lyoprotectants for *L. curvatus* via response surface methodology, identifying skim milk powder and sucrose as the most effective protective agents. Accordingly, skim milk powder was determined as a critical protectant, with an optimal concentration of 12%. As a polyol, sorbitol protects bacterial cells from dehydration damage and maintains viability during freeze-drying [26]. The results demonstrated that trehalose, lactose and skim milk powder possessed more prominent lyoprotective effects than sorbitol during vacuum freeze-drying. As typical disaccharides, trehalose and lactose can effectively replace water molecules surrounding microbial cells under dehydration stress. This substitution maintains the natural structure of the lipid bilayer on the cell membrane, prevents membrane damage and protein denaturation caused by ice crystal formation and water loss, and thus greatly improves cell survival. In addition, skim milk powder can form a dense protective film on the cell surface, which further buffers the damage from low temperature and dehydration. By contrast, although sorbitol also has certain protective capabilities, its molecular structure and water replacement efficiency are inferior to the above disaccharides, resulting in relatively weaker lyoprotective performance. The combined application of trehalose, lactose and skim milk powder achieves a synergistic protective effect, which accounts for the highest viability of lactic acid bacteria after freeze-drying. Excessive gluten relaxation caused by over-fermentation reduces dough gas retention capacity, decreasing steamed bun specific volume and height-diameter ratio. This observation is consistent with Tang et al. [27], who reported that increased LAB addition elevates steamed bun spreadability, further validating the balance between LAB dosage and dough rheological properties.

This research successfully screened superior LAB strains from natural sourdough, clarified the mechanism of LAB in improving dough quality and steamed bun flavor, and established an optimized vacuum freeze-drying preparation technology for LAB powder. The research results not only enriched the resource library of functional LAB strains for staple food processing but also provided a theoretical basis and technical support for the development of high-efficiency, stable, and standardized LAB starters. However, this study still has certain limitations: the interaction mechanism between multi-strain LAB in complex dough systems needs to be further explored; the long-term application effect and safety of the freeze-dried bacterial powder in actual industrial production require more pilot-scale verification; and the synergistic relationship between LAB and yeast in co-fermentation needs to be further optimized. Future research will focus on the development of compound starter cultures with stronger stability and functionality and conduct in-depth research on the application of LAB powder in different types of staple foods, so as to promote the modernization and industrialization of traditional fermented food processing.

## 5. Conclusions

This study developed an efficient and industrially feasible direct-vat-set lactic acid bacteria starter for steamed bun fermentation. The co-culture of *L. plantarum* and *S. thermophilus* displayed prominent synergistic effects with natural sourdough, effectively modulating dough acidification, refining gluten network architecture, and improving the texture, volumetric properties and sensory profiles of steamed buns. This compound fermentation strategy effectively mitigates the quality fluctuation and flavor deficiencies commonly observed in traditional sourdough fermentation. Through systematic optimization of centrifugation parameters and lyoprotectant composition, a robust preparation protocol for high-viability freeze-dried LAB powder was established. The optimized starter system achieves stable fermentation activity and controllable acidification, supporting consistent and high-quality steamed bun production. This work provides a practicable technical approach for the standardization and industrial upgrading of traditional fermented staple foods. The developed compound starter resolves the poor repeatability and uncontrollability of natural sourdough fermentation, presenting great potential for modern steamed bun manufacturing and offering a valuable reference for high-efficiency starter development in the baking industry.

## Figures and Tables

**Table 1:** Different dough compound proportion.

Dough Type	Sourdough Addition/%	LAB Suspension Addition/%	LAB	Suspension Mixing Ratio/*v*/*v*
Control	1	0	—	—
LP-Y	1	20	*Lactiplantibacillus plantarum*	—
PP-Y	1	20	*Pediococcus pentosaceus*	—
ST-Y	1	20	*Streptococcus thermophilus*	—
LP-PP-Y	1	20	*Lactiplantibacillus plantarum* *Pediococcus pentosaceus*	1:1
LP-ST-Y	1	20	*Lactiplantibacillus plantarum* *Streptococcus thermophilus*	1:1
ST-PP-Y	1	20	*Streptococcus thermophilus* *Pediococcus pentosaceus*	1:1
LP-ST-PP-Y	1	20	*Lactiplantibacillus plantarum* *Pediococcus pentosaceus* *Streptococcus thermophilus*	1:1:1

**Table 2:** Steamed buns sensory scoring criteria.

Project	Evaluation Standard	Score
Epidermal color	9–10 points for bright and normal color; 6–8 points for normal color but poor brightness; 3–5 points for dull color; 0–2 points for extremely poor color	10
Epidermal structure	14–15 points for smooth epidermis; 1–2 points deducted for each spot, bubble, or wrinkle	15
Internal structure	11–15 points for small and uniform pores; 6–10 points for uniform but large pores; 1–5 points for rough and uneven pores	15
Elasticity	11–15 points for quick rebound and complete recovery; 6–10 points for slow rebound but complete recovery; 1–5 points for no recovery	15
Taste	26–35 points for refreshing, non-sticky, and chewy; 16–25 points for moderate chewiness and non-sticky; 6–15 points for non-chewy and non-sticky; 0–5 points for non-chewy and sticky	30
Flavor	11–15 points for strong wheat flavor; 6–10 points for weak wheat flavor without off-odor; 0–5 points for off-odor	15

**Table 3:** Factors and levels of response surface methodology.

Level	A/%	B/%	C/%
−1	4	12	6
0	8	15	12
1	12	18	18

**Table 4:** Centrifugal recovery rate under different centrifugal conditions.

Group	Centrifugal Speed/*g*	Centrifugation Time/min	Viable Count Before Centrifugation/CFU/mL	Viable Count After Centrifugation/CFU/mL	Centrifugal Recovery Rate/%
1	4000	5	(4.60 ± 0.42) × 10^9^	(2.67 ± 0.04) × 10^9^	58.09 ± 0.75 ^f^
2	4000	10	(4.60 ± 0.42) × 10^9^	(4.03 ± 0.06) × 10^9^	87.61 ± 1.54 ^bc^
3	4000	15	(4.60 ± 0.42) × 10^9^	(4.13 ± 0.72) × 10^9^	89.78 ± 1.57 ^b^
4	6000	5	(4.60 ± 0.42) × 10^9^	(3.96 ± 0.27) × 10^9^	86.17 ± 2.00 ^bc^
5	6000	10	(4.60 ± 0.42) × 10^9^	(3.57 ± 0.09) × 10^9^	77.61 ± 2.01 ^e^
6	6000	15	(4.60 ± 0.42) × 10^9^	(4.47 ± 0.15) × 10^9^	97.17 ± 1.32 ^a^
7	8000	5	(4.60 ± 0.42) × 10^9^	(3.80 ± 0.20) × 10^9^	82.61 ± 2.34 ^d^
8	8000	10	(4.60 ± 0.42) × 10^9^	(4.21 ± 0.35) × 10^9^	91.49 ± 1.85 ^b^
9	8000	15	(4.60 ± 0.42) × 10^9^	(3.88 ± 0.38) × 10^9^	84.26 ± 3.12 ^cd^

Note: Different letters in the same column indicate significant differences between values (*p* < 0.05).

**Table 6:** Analysis of variance for response surface methodology experiments.

Source of Variation	Sum of Squares	Degrees of Freedom	Mean Square	*F* Value	*p* Value	Significance
Model	3984.63	9	442.74	59.80	<0.0001	***
A	920.42	1	920.42	124.32	<0.0001	***
B	83.08	1	83.08	11.22	0.0123	*
C	157.80	1	157.80	21.31	0.0024	**
AB	80.64	1	80.64	10.89	0.0131	*
AC	269.12	1	269.12	36.35	0.0005	***
BC	201.36	1	201.36	27.20	0.0012	**
A^2^	1004.77	1	1004.77	135.72	<0.0001	***
B^2^	415.62	1	415.62	56.14	0.0001	***
C^2^	622.36	1	622.36	84.06	<0.0001	***
Residual	51.82	7	7.40			
Lack of fit	30.39	3	10.13	1.89	0.2723	No significant
Pure error	21.43	4	5.36			
Total variation	4036.45	16				

Note: A = trehalose, B = lactose, C = skim milk powder; A *** indicates extremely significant (*p* < 0.001); ** indicates highly significant (*p* < 0.01); * indicates significant (*p* < 0.05).

**Table 7:** Results of storage stability of freeze-dried bacterial powder.

Sample	Viable Cell Count/CFU/g	Moisture Content/%	Cell Survival Rate/%
Initial powder	4.0 × 10^9^	1.8	95
Storage powder	3.3 × 10^9^	2.1	78

## Data Availability

The datasets used during the current study are available from the corresponding author on reasonable request.

## References

[1] Akinyemi M.O., Ogunremi O.R., Adeleke R.A., Ezekiel C.N. (2024). Probiotic potentials of lactic acid bacteria and yeasts from raw goat milk in Nigeria. Probiotics Antimicro. Proteins.

[2] Sorée M., Kolypczuk L., Hadjiev E., Lozach S., Verrez-Bagnis V., Delbarre-Ladrat C., Heath D.H., Passerini D. (2023). Screening of marine lactic acid bacteria for inhibition and application to depuration in Pacific oysters. J. Appl. Microbiol..

[3] Ma S., Wang Z., Guo X.F., Wang F.C., Huang J.H., Sun B.H., Wang X.X. (2021). Sourdough improves the quality of whole-wheat flour products: Mechanisms and challenges-A review. Food Chem..

[4] Warburton A., Silcock P., Eyres G.T. (2022). Impact of sourdough culture on the volatile compounds in wholemeal sourdough bread. Food Res. Int..

[5] Sha H.Y., Wang Q.Q., Li Z.J. (2023). Comparison of the effect of exopolysaccharide-producing lactic acid bacteria from sourdough on dough characteristics and steamed bread quality. Int. J. Food Sci. Tech..

[6] Sun X.Y., Wu S.M., Li W., Koksel F., Du Y.F., Sun L., Fang Y., Hu Q.H., Pei F. (2023). The effects of cooperative fermentation by yeast and lactic acid bacteria on the dough rheology, retention and stabilization of gas cells in a whole wheat flour dough system-A review. Food Hydrocoll..

[7] Yaqoob S., Liu H.M., Liu M.H., Zheng M.Z., Awan K.A., Cai D., Liu J.S. (2022). The effect of lactic acid bacteria and co-culture on structural, rheological, and textural profile of corn dough. Food Sci. Nutr..

[8] Ren H., Zentek J., Vahjen W. (2019). Optimization of production parameters for probiotic *Lactobacillus* strains as feed additive. Molecules.

[9] Lee S.B., Kim D.H., Park H.D. (2016). Effects of protectant and rehydration conditions on the survival rate and malolactic fermentation efficiency of freeze-dried JH287. Appl. Microbiol. Biot..

[10] Wang Q.Y., Zhang H.F., Zhu W.Z., Li C.M., Xu Y., Ding X.L., Zhou X.Y. (2022). Physicochemical properties and nutritional quality of pre-fermented red bean steamed buns as affected by freeze-thaw cycling. Int. J. Food Prop..

[11] (2009). Sensory Analysis—General Guidance for the Design of Test Rooms.

[12] Chen D., Cheng Q., Xia X.X., Chen S.H., Shao C.Q., Li J., Wang J.S., Wu X.Q. (2021). The state of water is associated with the viability and acidification capacity of *Lactobacilli* in frozen sourdough. Czech J. Food Sci..

[13] Cardinali F., Garofalo C., Reale A., Boscaino F., Osimani A., Milanovic V., Taccari M., Aquilanti L. (2022). Liquid sourdough from stone-ground soft wheat flour: Development and exploitation in the breadmaking process. Food Res. Int..

[14] Cizeikiene D., Jagelaviciute J., Stankevicius M., Maruska A. (2020). Thermophilic lactic acid bacteria affect the characteristics of sourdough and whole-grain wheat bread. Food Biosci..

[15] Wu S.M., Peng Y.L., Xi J.Z., Zhao Q.Y., Xu D., Jin Z.Y., Xu X.M. (2022). Effect of sourdough fermented with corn oil and lactic acid bacteria on bread flavor. LWT Food Sci. Technol..

[16] Oshiro M., Tanaka M., Momoda R., Zendo T., Nakayama J. (2021). Mechanistic insight into yeast bloom in a lactic acid bacteria relaying-community in the start of sourdough microbiota evolution. Microbiol. Spectr..

[17] Xu D., Zhang Y., Tang K.X., Hu Y., Xu X.M., Gänzle M.G. (2019). Effect of mixed cultures of yeast and *Lactobacilli* on the quality of wheat sourdough bread. Front. Microbiol..

[18] Wang X.Y., Huangfu X.Y., Zhao M.Y., Zhao R.Y. (2023). Chinese traditional sourdough steamed bread made by retarded sponge-dough method: Microbial dynamics, metabolites changes and bread quality during continuous propagation. Food Res. Int..

[19] Zhang G.H., Zhang W.Z., Sadiq F.A., Arbab S.H., He G.Q. (2019). Microbiota succession and metabolite changes during the traditional sourdough fermentation of Chinese steamed bread. CyTA J. Food.

[20] Rolim M.E., Fortes M.I., Von Frankenberg A., Duarte C.K. (2024). Consumption of sourdough bread and changes in the glycemic control and satiety: A systematic review. Crit. Rev. Food Sci..

[21] Adepehin J.O., Enujiugha V.N., Badejo A.A., Young G.M., Odeny D.A. (2023). Physicochemical and sensory attributes of gluten-free sourdough breads produced from underutilised African cereal flours and flour blends. Int. J. Food Sci. Technol..

[22] Chen B.Y., Wang X.Y., Li P.Z., Feng X.X., Mao Z.H., Wei J.J., Lin X., Li X.W., Wang L. (2023). Exploring the protective effects of freeze-dried under optimized cryoprotectants formulation. LWT Food Sci. Technol..

[23] Rakchai N., Maneerat S. (2022). Improved Survival of Freeze-Dried *Lactobacillus pentosus* SY130 and Applied as a Co-culture Starter with *Lactobacillus plantarum* KJ03 for Fermenting Stink Bean (Sataw-Dong). Indian J. Microbiol..

[24] Cheng Z.Y., Yan X., Wu J.Y., Weng P.F., Wu Z.F. (2022). Effects of freeze drying in complex lyoprotectants on the survival, and membrane fatty acid composition of *Lactobacillus plantarum* L1 and *Lactobacillus fermentum* L2. Cryobiology.

[25] Gul L.B., Con A.H., Gul O. (2020). Storage stability and sourdough acidification kinetic of freeze-dried N19 under optimized cryoprotectant formulation. Cryobiology.

[26] Luangthongkam P., Blinco J.A., Dart P., Callaghan M., Speight R. (2021). Comparison of spray-drying and freeze-drying for inoculum production of the probiotic strain H57. Food Bioprod. Process..

[27] Tang X.J., Liu R.S., Huang W.N., Zhang B.L., Wu Y.X., Zhuang J., Omedi J.O., Wang F., Zheng J.X. (2018). Impact of in situ formed exopolysaccharides on dough performance and quality of Chinese steamed bread. LWT Food Sci. Technol..

